# Menstrual Cycle Phase Does Not Predict Political Conservatism

**DOI:** 10.1371/journal.pone.0112042

**Published:** 2015-04-29

**Authors:** Isabel M. Scott, Nicholas Pound

**Affiliations:** Division of Psychology, Department of Life Sciences, Brunel University London, Uxbridge UB8 3PH, United Kingdom; Brock University, CANADA

## Abstract

Recent authors have reported a relationship between women's fertility status, as indexed by menstrual cycle phase, and conservatism in moral, social and political values. We conducted a survey to test for the existence of a relationship between menstrual cycle day and conservatism.

2213 women reporting regular menstrual cycles provided data about their political views. Of these women, 2208 provided information about their cycle date, 1260 provided additional evidence of reliability in self-reported cycle date, and of these, 750 also indicated an absence of hormonal disruptors such as recent hormonal contraception use, breastfeeding or pregnancy. Cycle day was used to estimate day-specific fertility rate (probability of conception); political conservatism was measured via direct self-report and via responses to the "Moral Foundations” questionnaire. We also recorded relationship status, which has been reported to interact with menstrual cycle phase in determining political preferences.

We found no evidence of a relationship between estimated cyclical fertility changes and conservatism, and no evidence of an interaction between relationship status and cyclical fertility in determining political attitudes. Our findings were robust to multiple inclusion/exclusion criteria and to different methods of estimating fertility and measuring conservatism. In summary, the relationship between cycle-linked reproductive parameters and conservatism may be weaker or less reliable than previously thought.

## Introduction

The possibility that women might exhibit systematic perceptual, cognitive, emotional and/or behavioural changes across the menstrual cycle has been of interest to scientists for many years. Following an initial preponderance of null findings for changes in cognitive performance in particular [1], in recent years evidence has accumulated indicating that there are cyclic changes in a range of cognitive, emotional and sensory processes (for reviews see e.g. [2–4]). Studies have begun to examine behavioural implications of such cyclicity [5,6] and an intriguing recently published paper by Durante and colleagues [7], reported evidence of a relationship between menstrual cycle phase and political conservatism, suggesting that political ideology may be modulated by fluctuations in female reproductive hormone levels. If robust, this finding could have important implications, both practical and academic, given not just the within-individual endocrine changes that women experience over the menstrual cycle and across the lifespan but also in light of the existence of substantial intra- and inter-populational differences in ovarian steroid levels [8]. However, the robustness of this effect is currently uncertain. Attempted direct replications of Durante et al’s findings have produced equivocal results, with certain of their results being only partially replicable and others not replicable at all [9,10]. Conceptual replications (i.e. tests for the same effect, using different methods) moreover, are currently lacking altogether.

A great deal of work by evolutionary psychologists has examined shifts in female mating preferences and behaviour across the menstrual cycle (e.g. for review see [3,11,12]) and Durante et al [7] develop their central hypothesis as an extension of findings in this area. Specifically, they argue that since “ovulation leads women to be more open to short-term sexual relationships, ovulation also might alter women’s religious and political attitudes to facilitate such relationships” and specifically lead them to become “less religious and more liberal” (p. 1008). The authors additionally propose, however, that there should be an adaptive interaction between the effects of ovulation and relationship status, with married women becoming more *conservative* around ovulation, in order “to promote relationship stability, commitment, and security” (p. 1009). Consistent with these predictions, they report finding moderate/large effects of cycle phase on conservative socio-political attitudes, with single women becoming more liberal around ovulation, and the effect reversed among married women. The authors propose that these effects are adaptive, and of relevance to “universal” variation in liberal-conservative politics.

While the specific, directional predictions above are derived from an adaptationist theoretical perspective, the more general hypothesis that there may be *some kind of relationship* between menstrual cycle phase and political attitudes can be derived from other, empirical considerations. The idea that political attitudes might vary in some way across the menstrual cycle is quite plausible since, as noted above, evidence for cyclic changes has been reported for a broad range of cognitive, emotional and sensory processes [2,13,14] (see [15] for review) and for diverse behaviours (e.g. [5,6]). Some of these reported shifts, moreover, are for variables that may be correlates of political conservatism, such as disgust [16–19] (although see [20]), an emotion that likely plays a role in many moral judgements [21]. Given the wide range of topics to which moral and political attitudes apply, the evidence of both a wide-ranging reorientation of attitudes across the cycle, and of changes across the cycle in potentially mediating factors (e.g. disgust), it is reasonable to hypothesise that political attitudes might vary cyclically—even if this were epiphenomenal to other processes, rather than an evolved adaptation. Accordingly, the hypothesis of a relationship between menstrual cycle and political preferences does appear to be a reasonable one, and one that is supported by the findings of Durante et al [7]. Given the intriguing nature of the findings, their possible implications and potential to stimulate interesting new directions for research, but also the difficulty that other authors have had in replicating them, it is important to examine the extent to which they are generalizable and robust.

One problematic general issue for all investigations of menstrual cycle effects is that researchers have a great deal of flexibility when it comes to defining both their independent and dependent variables and any exclusion criteria [22] (although see [23]). For example, there are numerous ways of specifying fertile and non-fertile phases of the cycle and also various methods for estimating when these occur. Whenever scientists are faced with a large number of ways in which they can test for effects, this raises an issue that has become known as the “researcher degrees of freedom” problem [24] which, while not implying any misrepresentation of data, can create uncertainty about the robustness of results [25]. This concern is particularly acute in light of questions raised by recent meta-analyses regarding the replicability of menstrual cycle effects on other behaviours (sexual preferences for masculinity) [26,27]. Moreover, the effect sizes reported by Durante et al [7] were much larger, and the degree of flexibility in political attitudes more substantial, than is typically seen in studies of voting behaviour [28]. Consequently, it is perhaps particularly important to examine whether the findings are generalizable and robust, or perhaps might be unique to particular populations, methodologies and analytic techniques.

In accordance with the above, the present study was designed to test whether there are changes in political conservatism across the menstrual cycle associated with changing conception risk. Although this was not an attempted replication of Durante et al’s study—our data were collected prior to the publication of Durante et al [7] and consequently our methods are not identical—we did collect data on relationship-status, and our data hence allowed us in addition to establish whether, if such changes occur, they are moderated by relationship status, as well as whether they are large or small, and robust to different testing methodologies.

We address these questions by

- testing for a general relationship between menstrual cycle phase and political attitudes, without specific predictions as to direction of effect (hypothesis H1)
- testing the directional predictions from Durante et al regarding the interaction between relationship status and menstrual cycle phase (hypothesis H2)
- reporting results using different measures of conservatism, and, as recommended by other authors [28], presenting our results under a variety of different treatments of the data.

## Methods

### Participants and procedure

Participants were women recruited via crowdflower.com with an advertisement requesting participants from the USA only. Crowdflower is a website that links non-US based researchers to online respondents via US-based recruitment sites. The large majority of these (97% in our survey) are recruited via the site Amazon M-Turk. The survey ran from December 2012 until February 2013, and participants completed the survey in return for a small payment.

Participants were asked to indicate whether they had regular menstrual cycles. This was defined as lasting between 25 and 35 days, for each of the last six cycles, following a commonly used convention [7,29]. 3570 women responded to a request for participants and answered this question. Analyses reported below are restricted to the 2213(62%) women who answered “yes” to this question.

These respondents had a mean age of 29.5 (*SD* = 8.1) and >99% were from the US (50 states represented). The majority of participants reported that they were white (74.0%) and indicated they had completed some level of tertiary education (87.4%). 55.7% reported a religious affiliation (39.0% Christian). Median household income was $20,000-$30,000 per year. 58.2% reported currently being in a committed relationship (married or cohabiting) and 40.1% reported being single (includes divorced, separated or widowed).

After initial questions (age, and country and state of current residence) respondents answered a series of questions on their moral and political attitudes, followed by details of their menstrual cycle, health and lifestyle factors including contraceptive use, and various socio-economic and demographic indices.

### Ethics statement

The research was approved by Brunel University Department of Psychology Research Ethics Committee. Participants read and completed the survey themselves, and completed the survey online.

### Measures

#### Risk of conception

Women who reported regular menstrual cycles were asked to state their normal cycle length and how many days have passed since the start of their last menses. Risk of conception (following intercourse on a given cycle day) was then estimated using data from Wilcox, Dunson, Weinberg, Trussell & Baird [30], following prior studies on cycle-phase influences on behaviour (e.g. [16]; see S1 File for details).

Individual estimates based on self-reported cycle-date may yield less accurate measures of fertility than those produced by using hormonal assays to identify the date of ovulation [31], since women may not always accurately recall the first day of their last menstrual period (LMP date) and in any case there is significant within and between female variability in the timing of ovulation in relation to onset of menses [32,33]. However, due to practical and financial considerations, it is generally possible to achieve much greater sample sizes with self-report methods.

There are several reasons that this should have had only a limited influence on our ability to identify fertility effects. Women’s recall accuracy for LMP date, while not perfect, is fairly good with 74% accurate to within 1 day, and 81% to within 2 days [34]. Moreover, when using continuous estimates of conception risk rather than dichotomizing women into fertile and non-fertile phases, the effects of small errors (± 2 days) will not be large since there is a (high) correspondence between estimates of conception risk from consecutive days [30], and also between estimates for women with regular and irregular cycles [30]. Consequently, fertility estimates generated from self-reported cycle date are likely to be correlated with actual risk of conception (although the exact strength of this correlation is not known; see S1 File for discussion). The reduction in accuracy associated with estimates of conception risk derived from self-report (versus hormone assay) data will inevitably weaken the magnitude of any associations between fertility and psychological variables that might exist, but that problem can be partially offset by the large sample sizes that self-report methods permit, giving sufficient power to detect weaker associations.

For our initial analyses (see Table 1), we used participants’ reported days since menses to generate continuous estimates of conception risk for the day of study participation (forward-counting method), and did not make adjustments for participants normal length of cycle. This is in accordance with the methods specified by Wilcox et al [30], who provide estimates of the probability of conception following intercourse on a given cycle day counting from onset of previous menses when the date of next menses is not known.

**Table 1:** Pearson Correlations between Risk of Conception and Conservatism.

	Correlation between conception risk^a^ and political conservatism^b^		
	*r*	*p*	*n*
All women^c^	.016	.668	748
Single women^d^	.068	.214	334
Partnered women^d^	-.023	.404	404

^a^ Risk of conception from a single act of intercourse, estimated from menstrual cycle day [30]. Cycle-day is days since last menses (forward-counting method).

^b^ Conservatism is a composite measure of right-wing/conservative self-placement, and responses to the Moral Foundations questionnaire.

^c^ Single and partnered women analysed together. Women were included in these analyses if they passed two data-quality checks and confirmed that they were not currently/recently using hormonal contraception, pregnant or breastfeeding.

^d^ Women were classified as single if they reported their status as single, divorced, separated or widowed, and partnered if married, cohabiting or in a long-term relationship.

An alternative to this method is to adjust the days since menses according to the woman’s normal cycle length (reverse-day counting method) and to use this adjusted day to estimate fertility. Wilcox et al’s [30] prospective conception risk tables based solely on days since onset of last menses are not designed to be used in this way, and such an approach lacks some important empirical support. However, this reverse-counting method has been widely advocated and used in related literature on menstrual cycle effects (see S1 File for discussion). Accordingly, results using this method are reported subsequently (Table 2).

**Table 2:** Pearson Correlations between Risk of Conception and Conservatism, Under Alternative Methods of Estimating Cycle Day.

Method of estimating cycle day	Correlation between conception risk and political conservatism		
	*r*	*p*	*n*
Forward-counting^a^	.016	.668	748
Reverse-counting^b^	-.005	.886	719

^a^ Forward-counting conception risk estimates are based on unadjusted days since last menses.

^b^ Reverse-counting estimates are based on days since start of last menses, adjusted according to reported normal length of cycle.

#### Exclusion criteria

For our initial analyses we applied only those exclusions most robustly associated with data quality. These include clear evidence of providing unreliable data, and sources of major disruption to cyclical hormone changes (pregnancy, breastfeeding, hormonal contraception). After applying these exclusions, our “core” sample size (n = 750) was still high, with estimated power to detect moderate or larger effects, as reported in Durante et al.[7], of >99% (see Table B in S1 File for power estimates).

Following these initial analyses, we applied a number of additional exclusions, using criteria more tentatively linked to ovarian suppression, or less commonly applied in prior studies. These include older age (>30), low body weight, weight loss, smoking, use of mood-altering substance and alcohol, and illness. We also present our results with fewer, or no exclusions applied. Further information regarding exclusion criteria is provided in S1 File.

#### Conservatism

Conservatism was measured via responses to the 20-question version of the Moral Foundations Questionnaire [35,36,37] (see S1 File for questions), and via self-reported right-wing and conservative political ideology (self-placement on left-right and liberal-conservative Likert scales). The Moral Foundations Questionnaire (MFQ) is derived from Moral Foundations theory [35–37], which proposes the existence of five universal psychological systems upon which moral ideology is based. These foundations are “care”, “fairness”, “ingroup loyalty”, “authority” and “purity”, of which three (“ingroup loyalty”, “authority” and “purity”) are reliably predictive of political conservatism [35,36], and one (“purity”) of sexual conservatism specifically.

In our sample, and consistent with findings from prior research [35,36], there were moderate or strong inter- correlations between scores on the three MFQ dimensions of interest (“ingroup loyalty”, “authority” and “purity”) and the two self-placement (left-right, liberal-conservative) Likert scales (all *r*(748)>.35, *p*<.0001; see S1 File and Figure A within that file for further details). Moreover, internal consistency for the 14 items (those contributing to the 3 MFQ dimensions and the 2 self-placement scales) together was high (Cronbach’s alpha = .87). We therefore averaged responses to these 14 questions as our measure of overall moral and political conservatism. For comparison, however, we also present our results with our measure broken down into its component parts (a Right-wing/Conservatism self-placement dimension, and a Conservative Moral Foundations (MFQ) dimension). In addition, because cyclical shifts have been proposed to relate to sexual conservatism in particular [7], we also present tests using the purity dimension alone (Table 3).

**Table 3:** Pearson Correlations between Risk of Conception and Conservatism, Under Alternative Treatments of the Dependent Variable.

Measure of conservatism^a^	Correlation between conception risk and political conservatism		
	*r*	*p*	*n*
Composite measure	.016	.668	748
Right-wing/ Conservative self-placement	.063	.087	748
Conservative Moral Foundations	-.0002	.995	748
Purity Moral Foundation	.018	.621	748

^a^ Conservatism is presented both as a composite measure, and broken down into components. The composite measure is an average of all questions asked on the topic of conservative ideology. “Right-wing/Conservative self-placement” refers to the average of two self-placement questions on Left-Right and Liberal-Conservative ideology. “Conservative Moral Foundations” refers to averaged responses to questions on those three of the five Moral Foundations (“ingroup loyalty”, “authority” and “purity”) that are reliably associated with political conservatism and right-wing ideology. “Purity Moral Foundation” refers to averaged responses to 4 questions on the Purity Moral Foundation, which concerns sexual morality specifically.

#### Other measures

Participants reported their marital status. As potential covariates of political attitudes, we also collected data on number of children, age, education, household income, personal income, religion and ethnicity. Participants also completed, as part of a separate parallel investigation, a questionnaire about hunger, and some additional questions about disgust and morality. There were around 100 questions in total and median time to complete the survey was 25 minutes.

### Tests

There are multiple potential approaches to testing for menstrual cycle effects on behaviour, most of which are defensible on some criterion or other, and too many to feasibly report in a single paper. Accordingly, we start by presenting the subset of these possible analyses that we feel to be most methodologically defensible, and then present a range of alternative approaches for comparison and transparency. We hence prioritise approaches which minimise researcher flexibility—for example, using a correlational analysis with all days of the cycle included to test for continuous associations between conception risk and conservatism, rather than a discrete comparison approach (comparing high vs low fertility cycle phases) with some data points excluded (although this latter approach is also presented for comparison in S1 File). While the decision regarding which further subsets to present are inevitably somewhat arbitrary, because we are reporting a null result we start by presenting the approaches that we anticipate to be most powerful. Where this is ambiguous (e.g. where there is a trade-off between different aspects of power) we start by presenting the approach that is most common or widely advocated in previous literature. Because all of our results are non-significant, we present unadjusted p-values and do not make corrections for multiple testing.

## Results

**Fig 1:**
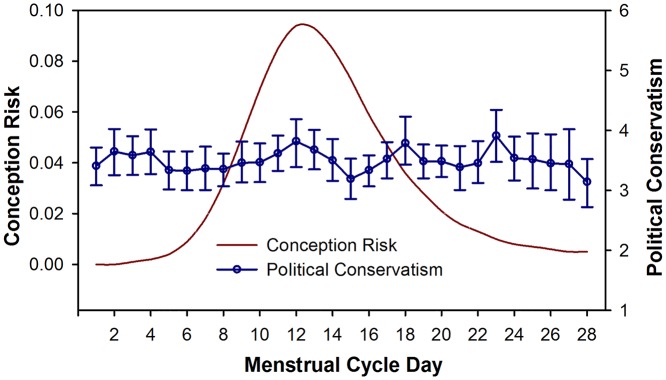
Political conservatism and risk of conception across the menstrual cycle. A high conservatism score indicates positive endorsement of conservatism. Conception Risk is an estimate of the probability of conception following intercourse on a given cycle day, counting from onset of previous menses (from Wilcox et al [30]). Plotted data is from women (n = 750) who passed data quality tests and confirmed that they were not currently/recently using hormonal contraceptives, pregnant or breastfeeding. Error bars are 95% confidence intervals.

For our initial analyses we tested for bivariate associations (Pearson’s *r*) between conception risk and our composite measure of political conservatism (hypothesis H1). In order to test the predictions derived from Durante et al.’s findings (hypothesis H2) we repeat these analyses with single and partnered women analysed separately, and then perform a *z*-test for comparisons between correlation coefficients for single and partnered women. Results are presented in Table 1 and patterns of conservatism across the menstrual cycle are plotted in Fig 1. For all measures of political attitudes (self-placement, Moral Foundations score, and composite measure), a high score is indicative of higher levels of conservatism.

There was no statistically significant association between estimated probability of conception (forward-counting) and the composite measure of political conservatism for the core sample as whole, *r*(746) = .016, *p* = .668, 95% *CI* [-0.05, 0.09], or with the participants split according to relationship status, single women: *r*(332) = .068, *p* = .214, 95% *CI* [-0.05, 0.18]; partnered women: *r*(402) = -.023, *p* = .404, 95% *CI* [-0.12, 0.08]. Conducting a *z*-test for differences between correlations in these two subgroups, we found no evidence of significant effects in the predicted direction—indeed, the trend was in the opposite direction to that implied by the findings from Durante et al., with paired women showing a weaker association between probability of conception and conservatism, and single women more conservatism, when fertile, z = 1.23, *p* = .219.

Using estimates of conception risk derived from the reverse-counting method did not change the pattern of results. The overall association between conception risk and conservatism remained non-significant, *r*(719) = -.005, *p* = .886, 95% *CI* [-0.08, 0.07]; see Table 2. Re-running analyses using alternative measures of conservatism (“right-wing/conservative self-placement”, “conservative moral foundations”, “purity moral foundation”) likewise yielded only non-significant results, all |*r*|<.07, *p*>.087; see Table 3. Finally, using a range of alternative exclusion criteria, we also failed to detect any significant relationships between estimated fertility and the composite measure of conservatism, all |*r*|<.09, *p*>.212; see Table 4.

**Table 4:** Pearson Correlations between Risk of Conception and Conservatism, After Applying Various Possible Exclusion Criteria.

Exclusion criteria, from least stringent (top) to most stringent (bottom)	Correlation between conservatism and risk of conception^e^		
	*r*	*p*	*n*
None	.024	.269	2124
Failed a data quality check^a^	.016	.561	1247
Previous criteria plus: Hormonal contraceptive use^b^, Pregnancy^c^ or breastfeeding^b^	.016	.668	748
Previous criteria plus: age>30	.063	.212	400
Previous criteria plus: Recent weight loss, Low body weight, Smoker, Recent use of mood-altering substances or alcohol^d^, Illness, Non-US resident	.084	.269	174

^a^ Data-quality checks included confirmation in two questions that menstrual cycle is typical length (between 25and 35 days), and less than one day’s discrepancy in responses to two framings of question about current day of menstrual cycle.

^b^ Current or last 3 months.

^c^ Current or last 3 years. Those uncertain about pregnancy status were excluded.

^d^ Use of mood-altering drugs or alcohol in last 72 hours, or antidepressants in last 3 months.

^e^ Forward-counted.

Re-running tests with Spearman rather than Pearson correlations produced qualitatively identical results, and there was no evidence of a relationship between risk of conception and any potential covariates of political conservatism such as age, number of children and socio-economic status variables (see S1 File)—all results were qualitatively the same after partialling out any effects of these variables. Finally, repeating our analyses using a discrete comparison approach—that is, comparing responses at “high” and “low” fertility phases of the cycle, as defined in Durante et al [7]—also did not yield any statistically significant associations between menstrual cycle phase and measure of conservatism (see S1 File for details).

## Discussion

In our data, we found no evidence of an association between menstrual cycle phase and political conservatism, either as a main effect, in the form of an interaction with relationship status, or with analyses carried out separately on the sample split according to relationship status. Rather than take a strong position on precisely which methods should be used to test for systematic menstrual cycle effects on the psychological variable of interest, we have instead repeated the analyses using many of the methods used in previously published work examining cyclic changes in other variables. This included using various methods of estimating and categorising conception risk, and using various exclusion and inclusion criteria to restrict and expand the sample under consideration.

Our results are therefore difficult to reconcile with those of Durante et al [7], particularly since we attempted the analyses using a range of approaches and exclusion criteria, including tests similar to those used by Durante et al, and our results were similar under all of them. Lack of statistical power does not seem a likely explanation for the discrepancy between our results and those reported in Durante et al, since even after the most restrictive exclusion criteria were applied, we retained a sample large enough to detect a moderate effect (as reported by Durante et al [7]) with very high probability (see Table B in S1 File). Moreover, our data quality was sufficient to detect other effects that have been documented by prior authors, such as relationships between different facets of moral and political conservatism [36], and between conservatism and socio-demographic factors such as age (see S1 File and Figure A within that file for details).

One factor that may partially explain the discrepancy is our different approaches to measuring conservatism and how the relevant questions were framed. Durante et al assessed social and fiscal political attitudes using items that related to specific contemporary policy issues that are likely widely understood to map onto the Democrat-Republican dimension around which political debate in the US is structured (e.g. abortion, same sex-marriage, taxation and social security policies). Moreover, additional questions in that study asked women to imagine voting for those party’s 2012 presidential candidates (Mitt Romney or Barack Obama). Given that there may be menstrual cycle shifts in person-preferences (see [22,23] for review), framing the liberal-conservative measures in this way, with references to specific individuals, could have generated apparent shifts in political attitudes secondary to changes in preferences for the specific candidates. In the present study, one way we assessed political conservatism was by measuring the more abstract moral foundations that, according to research building on moral foundations theory [35,36] underlie observed differences with political liberals and conservatives. Our measures therefore, are arguably less susceptible to preferences shifts associated with preferences for contemporary political figures. Consistent with this explanation, the direct replication attempted by Harris and Mickes [9], found partial support for Durante et al’s findings for voting behaviour, but no support for menstrual-cycle changes in social, religious and political attitudes.

However, these methodological differences seem unlikely to fully explain the discrepancy between our results, since Durante et al also found effects for variables that are unrelated to specific persons, such as religiosity and social conservatism. Moreover, it seems unlikely that Durante et al.’s measures simply captured some particularly malleable facet of political conservatism that we did not, given the replication failures reported by Harris and Mickes. One further possibility is that differences in responses to our survey and the other surveys discussed here [7,9,10] are attributable to variation in the samples surveyed. While all were recruited from the same source (MTurk), recent research has highlighted some potential issues with MTurk data which could result in inter-sample variation in responses [38]. These issues include inter-participant communication and participation in related studies following debriefing, both of which could, in principle, yield either false-positive or false-negative findings, particularly when multiple studies are run in close succession [38].

In summary, our data offer little support for the proposal that there is a substantial, politically significant, effect of menstrual cycle phase on political attitudes. It is nevertheless possible that there exists a relationship between these variables, and that we were unable to detect it. This might be due to the effect being small, or the measurement error associated with estimating fertility from self-reported cycle date being large. As outlined above, there is little reason to suppose that such errors are sufficient to mask a large (i.e. politically important) effect when tested in a large sample, but further research may help to clarify this issue. Ideally, this research would incorporate questions from both this study and those employed by Durante et al into a single survey, and would be replicated with longitudinal data and/or with conception risk estimates improved using urinalysis to confirm ovulation timing. If the finding of shifts in political attitudes across the cycle turns out to not be robust then a reappraisal of the rationale for expecting them to occur would be needed. Replication is of course essential for the advancement of scientific knowledge [39] and consequently it would be helpful for future research to continue to address the extent to which previously reported menstrual cycle effects on other behaviours are robust. We also echo prior authors’ calls for presentation of all data under a range of different analyses [28].

## Supporting Information

S1 File Supplemental materials. Contains detailed descriptions of measures and exclusion criteria, results of additional tests, additional tables and figures, and appendices reporting questionnaire items. **Table A, Number of participants meeting criteria for inclusion, according to different selection criteria. Table B, Power to Detect Effects of Different Sizes, on the Basis of Sample Size, After Applying Different Exclusion Criteria. Figure A, Correspondence between self-reported placement on a Liberal-Conservative political ideology scale, and endorsements of each of the five moral foundations from the Moral Foundations Questionnaire.** Mean score on MFQ dimension (±95% C.I.) at each point on the 7 point Liberal-Conservative self-placement scale for all women initially claiming regular samples and responding to these items (n = 2081). **Figure B, Histogram of reported number of days in a typical menstrual cycle. Figure C, Political conservatism across the menstrual cycle, split by relationship status of women.** A high score indicates high levels of conservatism. Plotted data is from women who passed data reliability tests (gave consistent answers across questions), and confirmed that they were not currently or recently using hormonal contraceptives, pregnant or breastfeeding. Error bars not shown due to extensive overlap across conditions. **Figure D, Political Conservatism in three phases of the menstrual cycle (±95% C.I.).** A high score indicates greater endorsement of conservative values. High and Low fertility phases are defined as days 7–14 and 17–25 respectively, after Durante et al (2013). PMS and menstruation phase is defined as days 1–6 and 26–28 inclusive. Plotted data is from women who passed data reliability tests (gave consistent answers across questions), and confirmed that they were not currently or recently using hormonal contraceptives, pregnant or breastfeeding.
(DOCX)
