# Supplementary material for: Menstrual Cycle Phase Does Not Predict Political Conservatism
Source: PLoS One. 2015 Apr 29;10(4):e0112042. doi: 10.1371/journal.pone.0112042 (PMC4414415; doi:10.1371/journal.pone.0112042)
Supplement: S1 File — Contains detailed descriptions of measures and exclusion criteria, results of additional tests, additional tables and figures, and appendices reporting questionnaire items. Table A, Number of participants meeting criteria for inclusion, according to different selection criteria. Table B, Power to Detect Effects of Different Sizes, on the Basis of Sample Size, After Applying Different Exclusion Criteria. Figure A, Correspondence between self-reported placement on a Liberal-Conservative political ideology scale, and endorsements of each of the five moral foundations from the Moral Foundations Questionnaire. Mean score on MFQ dimension (±95% C.I.) at each point on the 7 point Liberal-Conservative self-placement scale for all women initially claiming regular samples and responding to these items (n = 2081). Figure B, Histogram of reported number of days in a typical menstrual cycle. Figure C, Political conservatism across the menstrual cycle, split by relationship status of women. A high score indicates high levels of conservatism. Plotted data is from women who passed data reliability tests (gave consistent answers across questions), and confirmed that they were not currently or recently using hormonal contraceptives, pregnant or breastfeeding. Error bars not shown due to extensive overlap across conditions. Figure D, Political Conservatism in three phases of the menstrual cycle (±95% C.I.). A high score indicates greater endorsement of conservative values. High and Low fertility phases are defined as days 7–14 and 17–25 respectively, after Durante et al (2013). PMS and menstruation phase is defined as days 1–6 and 26–28 inclusive. Plotted data is from women who passed data reliability tests (gave consistent answers across questions), and confirmed that they were not currently or recently using hormonal contraceptives, pregnant or breastfeeding. (DOCX) [file pone.0112042.s001.docx]

Supplemental Material

# Methods

## Measures:

**Probability of conception.** Following prior authors [1–5], risk of conception was estimated using data on risk of conception from a single act of intercourse, for each day of the menstrual cycle [6], based on self-reported days since last menses. We used Wilcox et al’s [6] estimates for regular (versus irregular) cycles, due to the fact that only women who reported regular cycles were asked to report their cycle day, although the two estimates (irregular included/excluded) are very similar (see below).

The use of self-reported cycle-date to generate estimates may raise concerns about accuracy, given that some women may misremember their cycle date or have atypical-length menstrual cycles or phases. However, the extent to which this compromises the predictive utility of such estimates is unclear, and there are reasons to suppose that such estimates may nevertheless be reasonably well-correlated with actual fertility. Research indicates, for example, that most women are able to report their days since last menses fairly accurately (81% to within 2 days, 74% to within 1 day, and 56% on exact day) [7], figures that may be conservative for our data, where additional measures were taken to exclude individuals who were inconsistent across measures (i.e. whose responses diverged by more than 1 day between two phrasings of question). Moreover, there is temporal autocorrelation in fertility, so that the correlation between estimated probability of conception and estimated probability of conception two days afterwards is very high [6]. Finally, the correlation between the fertility estimates when women with irregular cycles are included/excluded is also very high [6], indicating that the existence of individual variation in typical or current cycle length need not undermine the aggregate relationship between cycle day and fertility. Although Wilcox et al [6] provide empirically based estimates of conception risk when cycle day is established with reference to a date of previous menstrual onset recorded using a prospective diary approach, evidence is lacking for the strength of the association between conception risk and cycle day estimates from retrospective recollection of LMP date in cross-sectional studies. Consequently, at present it is difficult to establish whether the reduction in accuracy associated with self-report (versus hormonal validation) is offset by increased sample size.

Forward versus backwards counting. Past research has commonly used one of two alternative methods for generating estimates of fertility from self-report data on a woman's current and typical menstrual cycle. Cycle day at the time of study participation is estimated by either “forward” counting from the date of onset for the last menstrual period, or by “backward”/“reverse” counting from the date of onset for the next menstrual period. In cross-sectional, rather than prospective/longitudinal studies, the LMP date is necessarily based on recollection while the next menstrual period date is projected based on self-report information on typical cycle length. Consequently, the former approach simply treats the day-of-cycle as the number of days that has elapsed since onset of last menses, while the latter adjusts this value according to typical cycle length. Specifically, with reverse-counting it is normally assumed that ovulation occurs 14 days prior to the end of a typical menstrual cycle and accordingly days elapsed since onset of last menses are converted to days until, or since, predicted day of ovulation. These estimates are then often mapped onto a standard 28 day cycle with ovulation predicted to occur on day 14. This method adjusts cycle-day upwards for women who report shorter cycles, and downwards for women reporting longer typical cycles [3]. In our sample, the most commonly reported cycle length was 28 days (40% of participants).

Wilcox et al [6] provide estimates of the probability of conception following intercourse on a given cycle day counting from onset of previous menses when the date of next menses is not known (i.e. for use with the forward-counting method). Using adjusted cycle day values (from backwards counting) in conjunction with Wilcox et al’s [6] data to generate conception risk estimates is a method that lacks important empirical support. However, this method has been widely used in prior research and accordingly we include analyses based on this method to enable comparisons with previous studies. Researchers have argued that backwards-counting methods provide a better index of fertility and hormonal status, due to lower variation in the length of the luteal phase (late cycle) than the follicular phase of the cycle [3–5,8]. However, the popular view that luteal phase lengths are relatively fixed at 14 days is not strongly supported by longitudinal studies which have identified significant variability in luteal phase durations [9–11] with 95% confidence intervals for luteal phase duration typically being around 9-18 days. Using the reverse-counting method has the additional disadvantage of requiring women to report two parameters accurately (normal cycle length and days since last menses), rather than just one (days since last menses), thus inflating the errors associated with misreporting. This is an important concern given that researchers (e.g. [12]) have reported poor recall of these parameters, and in particular that there is often a high level of confusion among respondents about the difference between cycle length and period of menstrual bleeding. This confusion was apparent in our own data: despite the fact that our cycle-length question was worded so as to make the distinction explicit (see appendix A), a substantial minority of women appeared to interpret the question about cycle length as pertaining to menstrual bleeding, as was evident in a clearly bimodal distribution of data with the first mode peaking at five days and the second peaking at 28 (see Figure B).

In accordance with the considerations above, our initial tests report results for forward-counted cycle day estimates, but we also report results for reverse-counted estimates, for comparison. We also start by excluding women reporting unusual cycle lengths, although we also present results without these exclusions, for comparison. We define unusual cycle lengths as being outside of the 25-35 day range, on the basis that this indicates inconsistency with our initial inclusion question. With exclusions in place for women who reported unusual cycle lengths and recent major hormonal disrupters, the correlation between estimates of conception risk using forward and backwards counting was high, *r*(716) = .83, *p* < .0001, suggesting that the two methods are likely to produce similar results.

In addition, we applied a discrete comparison approach, as in Durante et al [1] for the core sample of 750 women who reported regular cycles between 25-35 days in length, provided evidence of reliability in self-reported cycle date, and the absence of hormonal disruptors. Using each participant’s normal cycle length and number of days since the onset of previous menses, we used the established reverse-cycle day method to estimate the day of ovulation, and then categorize women in high fertility (the 8 days up to an including the day of ovulation, equivalent to cycle days 7-14 in a 28 day cycle) and low fertility (the 3^rd^ to 11^th^ days after ovulation, equivalent to cycle days 17-25 in a 28 day cycle) groups at the time of study participation.

**Conservatism.** Conservatism was measured via responses to 12 items within the 20-item Moral Foundations Questionnaire (MFQ-20) and also via self-placement on left-right and liberal-conservative Likert scales. The MFQ is an instrument that was developed to measure both inter and intra-cultural differences and similarities in moral attitudes. It is based on Moral Foundation Theory, which proposes the existence of (at least) five psychological systems upon which moral ideology is based, namely “care”, “fairness”, “ingroup loyalty”, “authority” and “purity”. These systems are proposed to be “universally available, but variably developed” [13].

Two of these proposed foundations (“care” and “fairness”) have been found to be valued by individuals of all political persuasions, while the other three (“ingroup loyalty”, “authority”, and “purity” – each assessed by 4 items on the MFQ-20) are valued more highly by political conservatives [14,15]. This finding has been replicated among participants from a range of countries and socio-economic backgrounds [13], suggesting that the MFQ may be more suitable for tests of universalist hypotheses regarding political conservatism than more culture-specific instruments. The MFQ has also been reported to have high internal and external validity [13].

Liberal-conservative and left-right self-placement Likert scales, while relatively simple measures, have also been shown to have reasonable validity and to be predictive of a number of explicit and implicit ideological constructs (see [16]), although as stand-alone items they have limited breadth and are subject to measurement error [16].

For the women in our sample, participants’ endorsement of each of MFQ foundations was calculated as their average score across the four questions from the MFQ-20 relevant to that foundation. Consistent with prior research, internal consistency was good for the loyalty (α = 0.625), authority (α = 0.684) and purity (α = 0.807) dimensions for our core sample (n = 750). Moreover, scores on these three dimensions were moderately correlated with each other (all *r* > 0.525; *p* < 0.0001) and with both conservative and right-wing self-placement scores, all *r* > .35, *p* < .0001. In contrast, scores on the loyalty, authority and purity dimensions were only weakly correlated with scores on the care and fairness dimensions, all | *r* | < .121, which in turn where only weakly (negatively) correlated with conservative or right-wing self-placement, all | *r* | < .111; see Figure A. Conservative and right-wing self-placement scores were also strongly correlated with each other, *r*(749) = .77, p <. 0001.

Accordingly, we created composite political conservatism scores by taking the average of 14 items, i.e. the 12 MFQ items assessing ingroup loyalty, authority and purity, and the 2 self-placement Likert scales assessing liberal-conservative and left-right self-identification. For comparison, however, we also report results for responses to the self-placement questions alone (averaged to produce a “Right-wing/Conservative self-placement” dimension), and for the MFQ (averaged responses to the 12 questions regarding ingroup loyalty, authority, and purity) questions alone. Finally, because cyclical shifts have been hypothesised to relate to mating strategies [1], we also present tests using the purity dimension alone (averaged scores across the four MFQ questions on this topic), as this relates to sexual behaviour specifically (see appendix B for the full list of questions, table 3 for results).

## Exclusion criteria:

Authors studying menstrual cycle effects on women’s behaviours have applied a wide and variable range of exclusion criteria when performing analyses [17]. While most of these are defensible on some ground or other, each involves trading-off sample size against data quality, and it is therefore often unclear whether the resulting tests will have increased or reduced power to detect true effects.

Accordingly, our initial analyses apply only those exclusion criteria that we anticipate to be most likely to result in increased power; that is, criteria for which there is strong evidence, or a strong *a priori* reason, to expect that cycle-phase effects will not be detectable in women who exhibit these criteria. These criteria include clear evidence of providing unreliable data, and factors for which there is very robust evidence of association with major disruption to ovulation and cyclical hormone changes (pregnancy, breastfeeding, hormonal contraception). Factors that are more tentatively or contingently associated with such disruption, and/or which tend to produce only minor disruption, are applied as additional exclusion criteria in subsequent tests, as described below and shown in table 4. For comparison, we also present results with fewer exclusion criteria applied.

Unreliable data. Although self-reported data on menstrual cycle parameters have been reported to be unreliable [18], authors commonly fail to include reliability checks when testing for effects of cycle phase on behaviour. To ensure that our data was of similar or higher quality than that reported in related studies, we incorporated two checks for consistency. First, women were asked to state the calendar date of the start of their last period (in addition to stating the number of days that had elapsed since the beginning of last menses), and a divergence of more than one day^[[1]](#footnote-1)^ between these two answers was treated as grounds for exclusion. Of the 2213 respondents reporting regular menstrual cycles and providing cycle day information, 1700 (76.8%) provided responses to these two questions that diverged by < 1 day.

Second, since initial recruitment materials asked for participants with typical cycles of 25-35 days in duration, we excluded participants who then went on to report a cycle length outside this range indicating inconsistent responses. Moreover, and as noted above, most scores outside this range were of a value that indicated a misinterpretation of the question (e.g. thinking duration of menses = cycle length), so can be assumed with fairly high confidence both to be erroneous, and to provide poor estimates of cycle-day when using the reverse-counting method. Of the 2213 respondents initially reporting regular menstrual cycles lasting 25-35 days and providing cycle day information, 622 (28.1%) subsequently reported that their usual cycle lengths was outside the day range.

Major hormonal disruptors. Hormonal contraceptive use, pregnancy and breastfeeding are all robustly associated with disruption to the cyclical shifts in ovarian steroids and fertility [19,20]. Whilst most published papers on menstrual cycle and behaviour exclude contraceptive users, some fail to ask about pregnancy and only a minority exclude women who are currently breastfeeding. In order to ensure that our data was of similar or higher quality to these papers, we classified respondents as experiencing disruption to cyclical hormonal shifts if they reported any of these factors; i.e. if they failed to confirm that they were not using hormonal contraception (current or within three months, including patches and injections), pregnant (now or within three years), or breastfeeding (within three months).

Age**.** The probability that a cycle will be anovulatory increases with female age [21], and it has been proposed that including older women in analyses reduces power to detect ovulation effects [22]. However, for any exclusion criteria less than age 50, the power reduction associated with loss of data could in principle exceed that associated with including older, potentially non-ovulating, women in analyses. Thus, without empirical validation, the introduction of any particular age threshold is arbitrary, and increases problems associated with researcher flexibility [17]. We therefore present our results without an age exclusion initially, but for comparison, and because age is a very commonly applied exclusion criterion (see [22]), we repeat our analyses with an age exclusion criteria applied. We use a threshold of age 30, on the basis that this is the most widely used convention applied in prior studies [22].

Other exclusion criteria. A range of other factors have been used as exclusion criteria in prior studies, or associated with some degree of hormonal disruption and/or increased probability of anovulation. These include low body weight, weight loss, smoking, use of mood-altering substance and alcohol, and illness [21,23–26]. In addition to this, Durante et al [1] used non-US residence as an exclusion criterion. We therefore asked questions on all of these criteria, and re-ran our analyses with the relevant exclusions applied. Women were classified as having low body weight if they reported a weight and height corresponding to BMI < 18.5, in line with current World Health Organization classifications.

# Results

## Re-running tests with spearman correlations

While Pearson correlations are generally more powerful than Spearman correlations, and produce similar estimates for large samples, Spearman correlations can sometimes be more powerful, especially in the case of skewed data. As risk of conception is generally skewed (and indeed was skewed in our sample, i.e. skewness/standard error of skewness > 2), we therefore re-ran our tests with Spearman correlations. Using this method, there was no significant association between estimated probability of conception (forward-counting) and conservatism in the full sample meeting basic inclusion criteria, *r_s_*(746) = .001, *p* = .977, or in the sub-samples of single women, *r_s_*(332) = .06, *p* = .294, and partnered women: *r_s_*(402) = -.03, *p* = .498. The relationship between conception risk and conservatism likewise remained non-significant after re-running analyses using a reverse-counting rather than forward-counting method, *r_s_*(717) = -.007, *p* = .854, and re-running analyses using alternative measures of conservatism (“right-wing/conservative self-placement”, “conservative moral foundations”, “purity moral foundation”; all | *r_s_* | < .05, *p* > .179). Finally, using the range of alternative exclusion criteria listed in the main text, we also failed to detect any significant relationships between estimated fertility and conservatism, all | *r_s_* |< .054, *p* > .279.

## Re-running tests with controls for potential confounds

As potential covariates of political attitudes, we also collected data on number of children, education, household and personal income, age, religion, and ethnicity, as well as on relationship status. Wording of questions and answer categories are shown in appendix A.

Political conservatism was positively associated with age, r(748) = .283, p < .0001, and number of children, r(729) = .322, p < .0001 but negatively associated with highest education level, r_s_(746) = -.126, p < .001.. Political conservatism scores were also higher among participants who identified themselves as having a religious affiliation, *F*(1,718) = 180.73, *p* < .0001, partial eta-sq = .20, and as being in a relationship, *F*(1,738) = 16.16, *p* < .0001, partial eta-sq = .021.

While there is no theoretical reason, of which we are aware, to expect that any of these variables would moderate or mediate the effects of female reproductive hormones on political preferences, it is nevertheless possible that these variables could predict women’s likelihood of taking the survey at different times of the menstrual cycle - for example, older women might be over- or under-represented at certain points in the cycle, since there are changes in the length of the menstrual cycle with age [27]. This possibility has been highlighted in the context of explaining possible false-positive findings [28], but in principle could also generate false-negative findings by cancelling out true-positives.

To check for such effects, we tested for significant relationships in the core sample of 750 (i.e. using forward-counting of cycle-day and exclusions for poor data quality and major hormonal disruptors) between risk of conception at the time of study participation and the above variables. There was no evidence of a significant relationship between probability of conception and any of the demographic or socioeconomic variables (age, children, education, household income, personal income: Pearson correlations: all | r | < .05, p > .228; Spearman correlations: all | r_s_ | < .04, p > .312) or any of the discrete variables (religion, ethnicity, relationship status: all F < .55, p > .737). These findings indicate that that the potential confounds listed above are unlikely to be obscuring significant relationships between probability of conception and conservatism in our data. To explore this further, however, we tested for partial correlations between risk of conception and conservatism, controlling for each of age, number of children, education, in turn, and performed GLM analyses with probability of conception entered as a covariate and each of religion, ethnicity and marital status entered in turn as fixed factors. These analyses did not produce any evidence of a statistically significant relationship between probability of conception and conservatism, all | r | < .017, p > .643; all F < .564, p > .453). Taken together these finding indicate that the potential confounds we examined are unlikely to be obscuring significant associations between conception risk and conservatism in our data.

## Applying a discrete comparison approach

One potential reason for the discrepancy between our results and those reported by Durante et al [1] is that we employ a different testing methodology. We perform correlations, analyzing data from throughout the entire menstrual cycle, whereas Durante et al’s primary analyses employ a discrete comparison between “high” and “low” fertility phases, discarding other data. While some authors (e.g. [28]) advocate analysing all available data, both in general and for menstrual cycle studies specifically, others have suggested that fertility status on certain days of the cycle is ambiguous and difficult to assign [1,29], which could make the discrete comparison approach more powerful.

To check for a role of methodology, a GLM analysis was conducted, with conservatism as a dependent variable and cycle phase and relationship status as independent variables. Following Durante et al [1], for the core sample of 750 the high fertility phase was defined as days 7-14 (n = 209) and the low fertility phase as days 17-25 (n = 214). For consistency with Durante et al., cycle-day was calculated using reverse-counting. As previously, exclusions for poor data quality and major hormonal disruptors were employed.

The overall model approached statistical significance, *F*(3,419) = 2.56, *p* = .054, partial eta-sq = .018, and there was a significant main effect of relationship status, *F*(1,419) = 6.59, *p* = .011, partial eta-sq = .016. Partnered women reported more conservative values than single women on average (Single women: *M* = 3.37, *SD* = .86; Partnered women: *M* = 3.59, *SD* = .94). However there was no evidence of an effect of cycle phase, *F*(1,419) = .566, *p* = .452, partial eta-sq < .001, or of an interaction between cycle phase and relationship status, *F*(1,419) = 0.611, *p* = .435, partial eta-sq = .001.

The analysis was then repeated with women classified as belonging to *three* groups: high fertility, low fertility and premenstrual/menstruating (following recommendations from Gelman 2013 [28]; see Figure D). The high and low fertility groups were defined as above, the PMS/menstrual group consisted of women on days 1-6 and days 26-28 of their cycle (n = 209). Again, the overall model was statistically significant, *F*(5,623) = 3.65, *p* = .003, partial eta-sq = .029, and there was a significant effect of relationship status (single women: *M* = 3.34, *SD* = .87, partnered women: *M* = 3.63, *SD* = .94), *F*(1,623) = 15.72, *p* < . 0001, partial eta-sq = .025, but no effect of cycle phase, *F*(2,623) = .279 *p* = .757, partial eta-sq < .001, or cycle-phase-relationship-status interaction, *F*(2,623) = 1.03, *p* = .358, partial eta-sq = .003.

Thus, when using discrete comparison methods there was no evidence of an effect on political conservatism of cycle phase, or of an interaction between cycle phase and relationship status, irrespective of whether premenstrual/menstruating women were excluded from analyses.

# Additional references

1. Durante KM, Rae A, Griskevicius V. The fluctuating female vote: politics, religion, and the ovulatory cycle. Psychol Sci. 2013;24: 1007–16. doi:10.1177/0956797612466416

2. Haselton MG, Gangestad SW. Conditional expression of women’s desires and men's mate guarding across the ovulatory cycle. Horm Behav. 2006;49: 509–518.

3. Gangestad SW, Garver-Apgar CE, Simpson JA, Cousins AJ. Changes in women’s mate preferences across the ovulatory cycle. J Pers Soc Psychol. 2007;92: 151–163.

4. Eastwick PW, Finkel EJ. The evolutionary armistice: attachment bonds moderate the function of ovulatory cycle adaptations. Pers Soc Psychol Bull. 2012;38: 174–84. doi:10.1177/0146167211422366

5. Prokosch MD, Coss RG, Scheib JE, Blozis SA. Intelligence and mate choice: intelligent men are always appealing. Evol Hum Behav. 2009;30: 11–20.

6. Wilcox AJ, Dunson DB, Weinberg CR, Trussell J, Baird DD. Likelihood of conception with a single act of intercourse: providing benchmark rates for assessment of post-coital contraceptives. Contraception. 2001;63: 211–215.

7. Wegienka G, Baird DD. A comparison of recalled date of last menstrual period with prospectively recorded dates. J Womens Health (Larchmt). Mary Ann Liebert, Inc.  2 Madison Avenue Larchmont, NY 10538 USA; 2005;14: 248–52. doi:10.1089/jwh.2005.14.248

8. Pillsworth EG, Haselton MG, Buss DM. Ovulatory shifts in female sexual desire. J Sex Res. Routledge; 2004;41: 55–65. doi:10.1080/00224490409552213

9. Baird DD, McConnaughey DR, Weinberg CR, Musey PI, Collins DC, Kesner JS, et al. Application of a method for estimating day of ovulation using urinary estrogen and progesterone metabolites. Epidemiology. 1995;6: 547–50.

10. Cole LA, Ladner DG, Byrn FW. The normal variabilities of the menstrual cycle. Fertil Steril. 2009;91: 522–527.

11. Fehring RJ, Schneider M, Raviele K. Variability in the phases of the menstrual cycle. J Obstet Gynecol Neonatal Nurs. 2006;35: 376–84. doi:10.1111/j.1552-6909.2006.00051.x

12. Gangestad SW, Thornhill R. Menstrual cycle variation in women’s preferences for the scent of symmetrical men. Proc R Soc London Ser B-Biological Sci. 1998;265: 927–933.

13. Graham J, Nosek BA, Haidt J, Iyer R. Mapping the Moral Domain. J Pers Soc Psychol. 2011;

14. Haidt J, Graham J. When Morality Opposes Justice: Conservatives Have Moral Intuitions that Liberals may not Recognize. Soc Justice Res. Springer Netherlands; 2007;20: 98–116.

15. Graham J, Haidt J, Nosek BA. Liberals and conservatives rely on different sets of moral foundations. J Pers Soc Psychol. 2009;96: 1029–46. doi:10.1037/a0015141

16. Tybur JM, Merriman LA, Hooper AEC, McDonald MM, Navarrete CD. Extending the Behavioral Immune System to Political Psychology: Are Political Conservatism and Disgust Sensitivity Really Related? Evol Psychol. 2010;Volume 8(4: 599–616.

17. Harris CR. Shifts in Masculinity Preferences Across the Menstrual Cycle: Still Not There. Sex Roles. 2012;69: 507–515. doi:10.1007/s11199-012-0229-0

18. Taffe J, Dennerstein L. Retrospective self-report compared with menstrual diary data prospectively kept during the menopausal transition. Climacteric. 2000;3: 183–191. doi:10.1080/13697130008500099

19. Lewis PR, Brown JB, Renfree MB, Short R V. The resumption of ovulation and menstruation in a well-nourished population of women breastfeeding for an extended period of time. Fertil Steril. 1991;55: 529–36.

20. Fleischman DS, Navarrete CD, Fessler DMT. Oral contraceptives suppress ovarian hormone production. Psychol Sci. 2010;21: 750–2; author reply 753. doi:10.1177/0956797610368062

21. Vitzthum VJ. Evolutionary Models of Women’s Reproductive Functioning. Annu Rev Anthropol. Annual Reviews; 2008;37: 53–73.

22. Debruine L, Jones BC, Frederick DA, haselton MG, Penton-Voak IS, Perrett DI. Evidence for Menstrual Cycle Shifts in Women’s Preferences for Masculinity: A Response to Harris (in press) “Menstrual Cycle and Facial Preferences Reconsidered” . Evol Psychol. 2010;8(4): 768–775.

23. Frisch RE. The right weight: body fat, menarche and fertility. Proc Nutr Soc. Cambridge University Press; 1994;53: 113–129.

24. Jensen TK, Hjollund NHI, Henriksen TB, Scheike T, Kolstad H, Giwercman A, et al. Does moderate alcohol consumption affect fertility? Follow up study among couples planning first pregnancy. BMJ. 1998;317: 505–510. doi:10.1136/bmj.317.7157.505

25. Van Voorhis BJ, Dawson JD, Stovall DW, Sparks AET, Syrop CH. The effects of smoking on ovarian function and fertility during assisted reproduction cycles. Obstet Gynecol. 1996;88: 785–791.

26. Fleischman DS, Fessler DMT. Progesterone’s effects on the psychology of disease avoidance: Support for the compensatory behavioral prophylaxis hypothesis. Horm Behav. 2011;59: 271–275.

27. Brodin T, Bergh T, Berglund L, Hadziosmanovic N, Holte J. Menstrual cycle length is an age-independent marker of female fertility: results from 6271 treatment cycles of in vitro fertilization. Fertil Steril. 2008;90: 1656–1661.

28. Gelman A. The connection between varying treatment effects and the crisis of unreplicable research: A Bayesian perspective. J Manage. 2013;

29. DeBruine LM, Jones BC, Perrett DI. Women’s attractiveness judgments of self-resembling faces change across the menstrual cycle. Horm Behav. 2005;47: 379–383.

# Appendices

## Appendix A: Independent variables and potential covariates

***Questions about menstrual cycle, health and lifestyle factors***

- Do you have regular menstrual cycles? That is, have you had a cycle lasting between 25 and 35 days, for each of the last 6 cycles?

If you are female and are going through, or have gone through, menopause, please just check "No"

[Yes/No/Don’t know/Not applicable/Do not wish to say]

- **PLEASE READ THIS QUESTION CAREFULLY**

How many days does your menstrual CYCLE usually last?

That is, how many days from the START of one period to the START of the next one?

For most (but not all) women, this is between 20 and 40 days.

It is NOT the same thing as the length of your period, which for most women is around 5

days.

[number]

- What was the date on which your last menstrual period started? If you are not sure,

please enter your best guess.

[MM DD YYYY]

- How many days ago was that (when your last menstrual period started)?

[number]

- Are you currently using any hormonal contraceptives? This could mean a contraceptive pill, patches or injections.

[Yes/No/Don’t know/Not applicable/Do not wish to say]

- Is the contraception you are using in the form of a pill?

[Yes/No/Don’t know/Not applicable/Do not wish to say]

- If so, is your pill progesterone only?

[Yes/No/Don’t know/Not applicable/Do not wish to say]

- Many women who take a contraceptive pill will take the pill for 21 days, and then take a 7 day break. If you are currently using a contraceptive pill, did you take a pill today, or are you on a 7 day break?

[I took a pill today/I am on my 7 day break/This question does not apply to me/Do not wish to say]

- Have you used hormonal contraceptives in the last 3 months?

[Yes/No/Don’t know/Not applicable/Do not wish to say]

- Are you pregnant?

[Yes/No/Don’t know/Not applicable/Do not wish to say]

- Have you been pregnant or given birth within the past three years?

[Yes/No/Don’t know/Not applicable/Do not wish to say]

- Are you currently breastfeeding, or have you been within the last 3 months?

[Yes/No/Don’t know/Not applicable/Do not wish to say]

- How many biological children do you have? This means children that you have given birth to or fathered yourself – please do not include stepchildren or adopted children.

[number]

- Do you have any chronic physical or mental illnesses?

[Yes/No/Don’t know/Not applicable /Do not wish to say]

- Are you currently taking any medication?

[Yes/No/Don’t know/Not applicable /Do not wish to say]

- Are you currently taking antidepressant medication, or have you been within the last 3 months?

[Yes/No/Don’t know/Not applicable/Do not wish to say]

- Have you recently lost a significant amount of your body weight?

[Yes/No/Don’t know/Not applicable/Do not wish to say]

- Have you taken any mood-altering drugs in the past 72 hours?

[Yes/No/Don’t know/Not applicable/Do not wish to say]

- Have you consumed alcohol in the past 72 hours?

[Yes/No/Don’t know/Not applicable/Do not wish to say]

- Do you smoke cigarettes on a daily basis?

[Yes/No/Don’t know/Not applicable/Do not wish to say]

- How much sleep did you get last night, in hours?

[number]

- How much sleep do you get on an average night, in hours?

[number]

- How much do you weigh? lbs (pounds)

[number]

- What is your height? Feet/inches

[number]

***Questions about Socio-Economic Status and Demographics***

- What is the highest level of education that you have completed?

Less than High School

High School/GED

Some College

2 year college degree (Associates)

4year college degree (BA, BS)

Master's Degree

Doctoral Degree

Professional Degree (MD, JD)

Do not wish to say

- What is your household income?

Less than $10,000

$10,001 - $20,000

$20,001 - $30,000

$30,001 - $40,000

$40,001 - $50,000

$50,001 - $60,000

$60,001 - $70,000

$70,001 - $80,000

$80,001 - $90,000

$90,001 - $100,000

$100,001 - $110,000

$110,001 - $120,000

More than $120,000

Do not wish to say

- What is your own income?

Less than $10,000

$10,001 - $20,000

$20,001 - $30,000

$30,001 - $40,000

$40,001 - $50,000

$50,001 - $60,000

$60,001 - $70,000

$70,001 - $80,000

$80,001 - $90,000

$90,001 - $100,000

$100,001 - $110,000

$110,001 - $120,000

More than $120,000

Do not wish to say

- How would you describe your religious affiliation?

Protestant Christian

Roman Catholic

Evangelical Christian

Jewish

Muslim

Hindu

Buddhist

Other religion

Not religious

Do not wish to say

- How would you describe your race or ethnicity?

White, nonHispanic

AfricanAmerican

Hispanic

AsianPacific

Islander

Native American

Other

Do not wish to say

- What is your current marital status?

Single, never married

Cohabiting and/or in a longterm relationship

Married

Divorced or separated

Widowed

Other

Do not wish to say

## Appendix B: Dependent variables

***Moral Foundations Questionnaire – 20 question version***

Part 1: When you decide whether something is right or wrong, to what extent are the following considerations relevant to your thinking? [Likert scale: not at all relevant – extremely relevant]

1. Whether or not someone suffered emotionally
2. Whether or not some people were treated differently than others
3. Whether or not someone’s action showed love for his or her country
4. Whether or not someone showed a lack of respect for authority
5. Whether or not someone violated standards of purity and decency
6. Whether or not someone cared for someone weak or vulnerable
7. Whether or not someone acted unfairly
8. Whether or not someone did something to betray his or her group
9. Whether or not someone conformed to the traditions of society
10. Whether or not someone did something disgusting

Part 2: Please read the following sentences and indicate your agreement or disagreement: [Likert scale: strongly disagree – strongly agree]

1. Compassion for those who are suffering is the most crucial virtue.
2. When the government makes laws, the number one principle should be ensuring that everyone is treated fairly.
3. I am proud of my country’s history.
4. Respect for authority is something all children need to learn.
5. People should not do things that are disgusting, even if no one is harmed.
6. One of the worst things a person could do is hurt a defenseless animal.
7. Justice is the most important requirement for a society.
8. People should be loyal to their family members, even when they have done something wrong.
9. Men and women each have different roles to play in society.
10. I would call some acts wrong on the grounds that they are unnatural.

For both parts of the questionnaire, the questions relevant to each foundation are as follows:

Care: 1, 6 ; Fairness: 2, 7; Loyalty: 3, 8; Authority: 4, 9; Purity: 5, 10

***Ideological Self-placement questions***

1. In political matters, people talk of "the left" and "the right." How would you place your views on this scale, generally speaking? [Likert scale: Left – Right]
2. And what about on a scale going from Liberal to Conservative? [Likert scale: Liberal – Conservative]

# Tables

*Table A*

*Number of participants meeting criteria for inclusion*

| Inclusion Criteria | Number Meeting Cumulative Criteria |
| --- | --- |
| 1. Women recruited | 3570 |
| 1. Answered yes to "*Do you have regular menstrual cycles? That is, have you had a cycle lasting between 25 and 35 days, for each of the last 6 cycles?*" | 2213 |
| 1. Subsequently responded to question "*How many days does your menstrual CYCLE usually last?*" with a value 25-35 days | 1591 |
| 1. Provided calendar date for onset of last menses and number of days since last menses that were in agreement +/- 1 day. | 1260 |
| 1. Not pregnant now or in past 3 years (and not breastfeeding in past 3 months). | 1038 |
| 1. Not using hormonal contraceptives now or in past 3 months | 750 |

*Table B.*

*Power to Detect Effects of Different Sizes, on the Basis of Sample Size, After Applying Different Exclusion Criteria:*

|  |  | Power^a^ to detect effects of size | | |
| --- | --- | --- | --- | --- |
| Exclusion criteria, from least stringent (top) to most stringent (bottom) | Sample size^b^ | Small  (r = .1) | Medium  (r = .3) | Large  (r = .5) |
| None | 2213 | 0.997 | 1.000 | 1.000 |
| Failed data quality checks^c^ | 1260 | 0.946 | 1.000 | 1.000 |
| Previous criteria plus: Hormonal contraceptive use^d^, Pregnancy^e^ or breastfeeding^d^ | 750 | 0.785 | 1.000 | 1.000 |
| Previous criteria plus: age > 30 | 401 | 0.520 | 1.000 | 1.000 |
| Previous criteria plus: Recent weight loss, Low body weight, Smoker, Recent use of mood-altering substances or alcohol^f^, Illness, Non-US resident | 174 | 0.261 | 0.985 | 1.000 |

a Probability of detecting an effect of size r or greater, with a two-tailed Pearson correlation.

^b^ Number of women who were included in tests of the correlation between risk of conception and conservatism, as reported in table 4.

^c^ Data-quality checks included confirmation in two questions that menstrual cycle is typical length (between 25 and 35 days), and less than one day’s discrepancy in responses to two framings of question about current day of menstrual cycle

^d^ Current or last three months

e Current or last three years. Those uncertain about pregnancy status were also excluded.

^f^ Use of mood-altering drugs or alcohol in last 72 hours, or antidepressants in last three months

# Figures


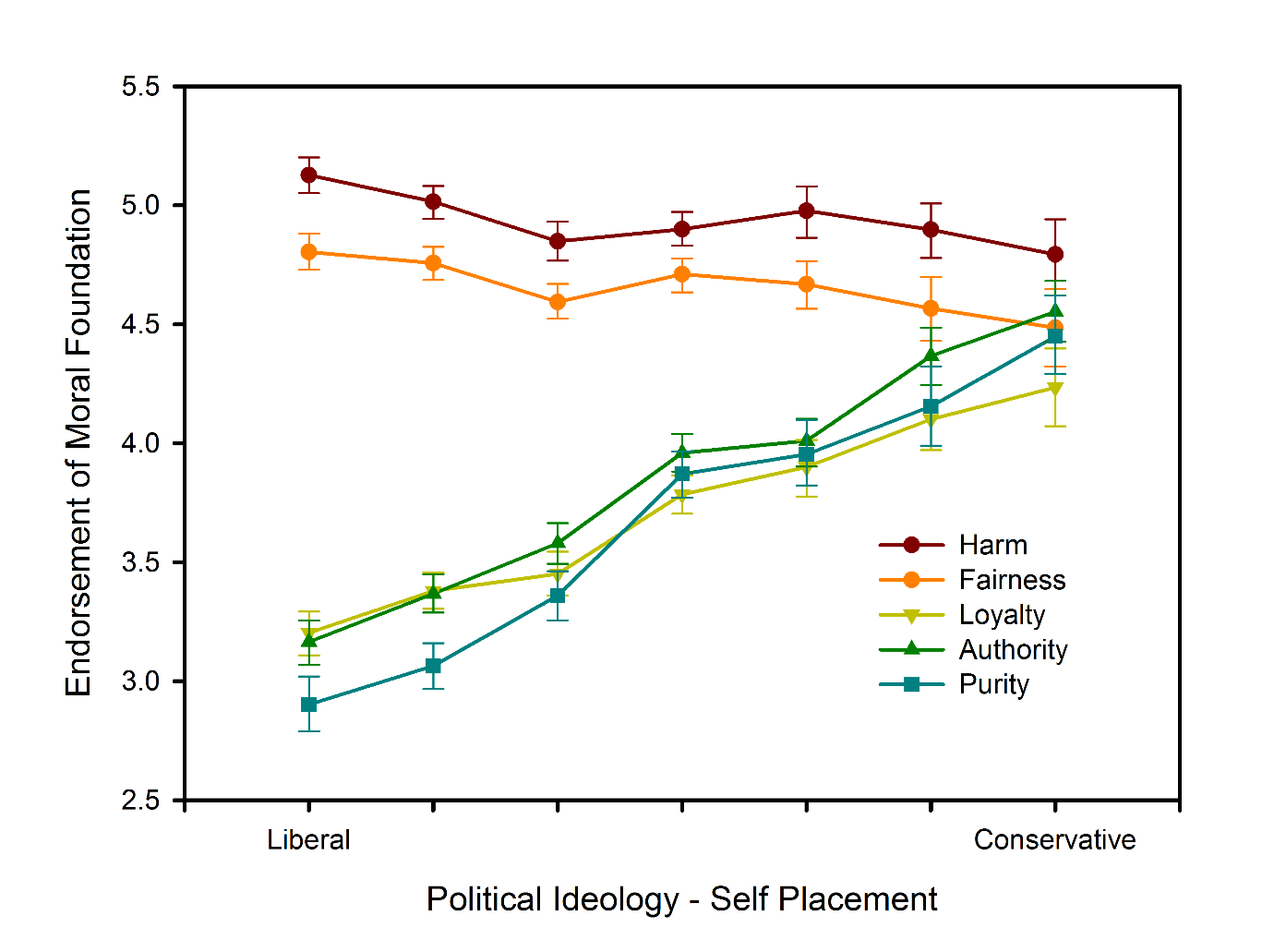


**Figure A. Correspondence between self-reported placement on a Liberal-Conservative political ideology scale, and endorsements of each of the five moral foundations from the Moral Foundations Questionnaire. Mean score on MFQ dimension (±95% C.I.) at each point on the 7 point Liberal-Conservative self-placement scale for all women initially claiming regular samples and responding to these items (n = 2081).**


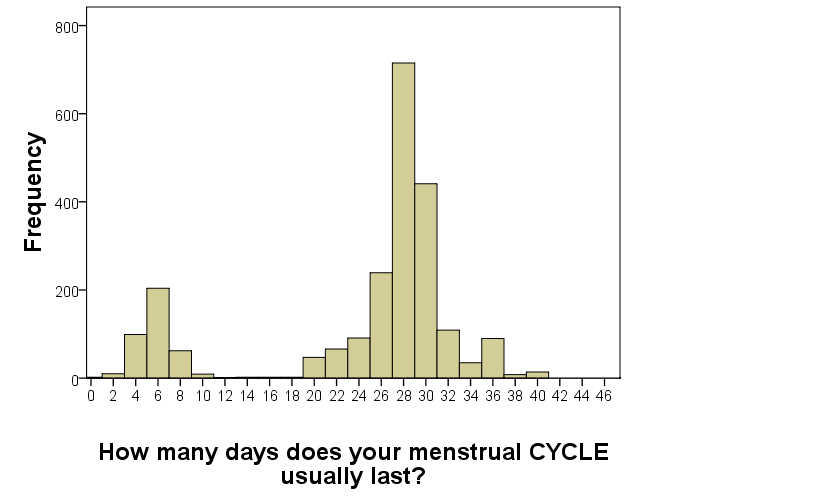


**Figure B. Histogram of reported number of days in a typical menstrual cycle**


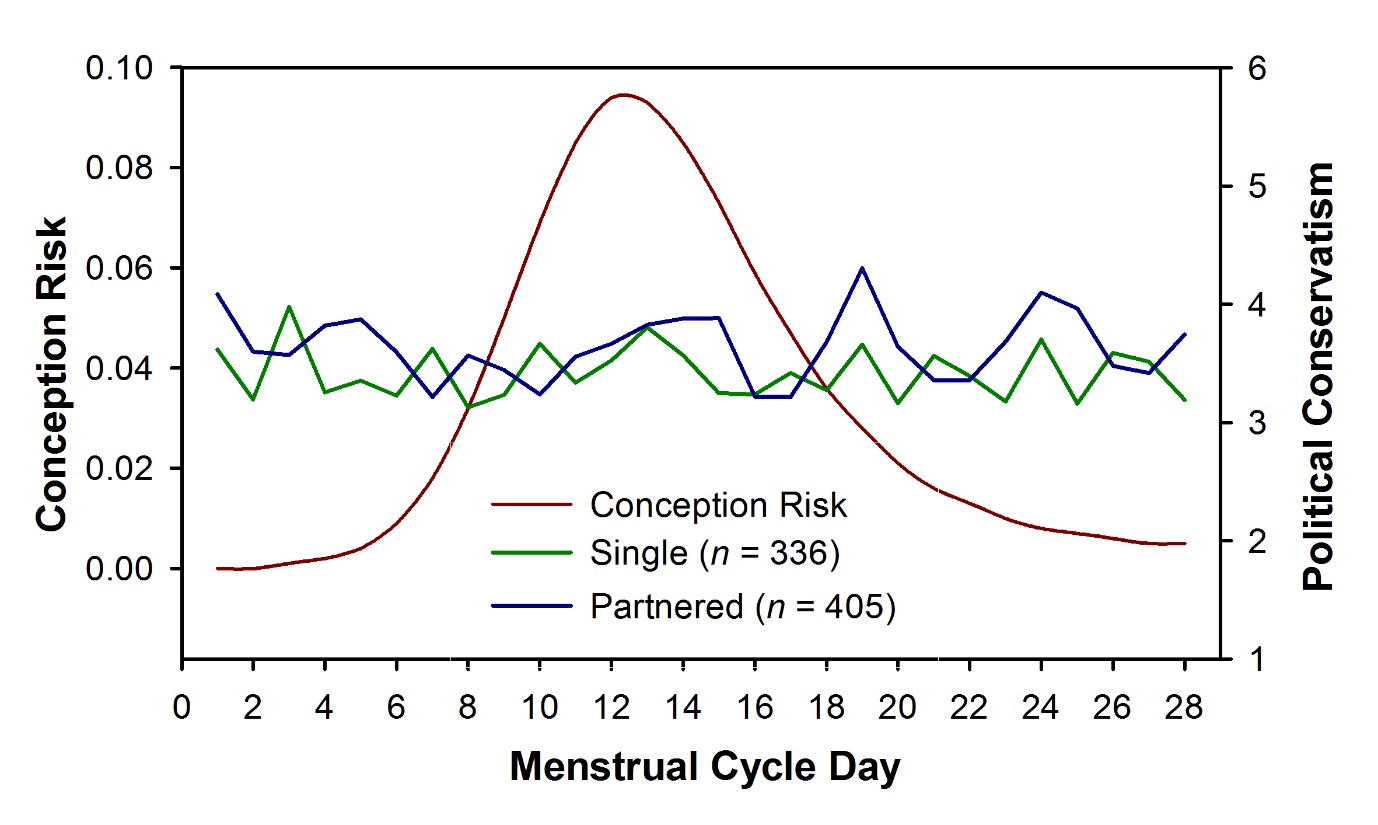


**Figure C. Political conservatism across the menstrual cycle, split by relationship status of women.** A high score indicates high levels of conservatism. Plotted data is from women who passed data reliability tests (gave consistent answers across questions), and confirmed that they were not currently or recently using hormonal contraceptives, pregnant or breastfeeding. Error bars not shown due to extensive overlap across conditions.


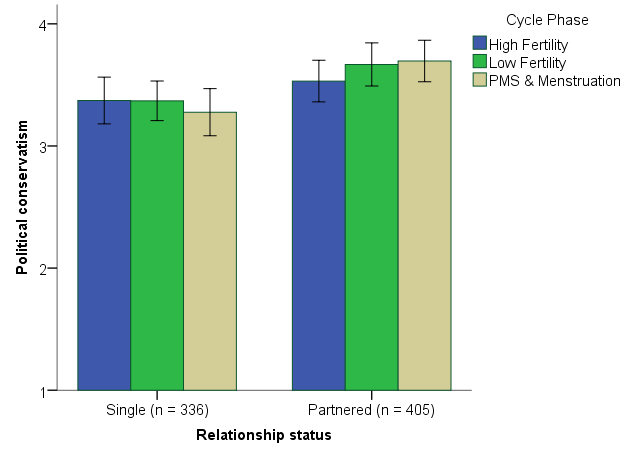


**Figure D. Political Conservatism in three phases of the menstrual cycle (±95% C.I.).** A high score indicates greater endorsement of conservative values. High and Low fertility phases are defined as days 7-14 and 17-25 respectively, after Durante et al (2013). PMS and menstruation phase is defined as days 1-6 and 26-28 inclusive. Plotted data is from women who passed data reliability tests (gave consistent answers across questions), and confirmed that they were not currently or recently using hormonal contraceptives, pregnant or breastfeeding.

1. A divergence of one day was not considered grounds for exclusion, as this could be attributable to time-zone differences between participants and researcher, and/or differences in participant definition of a day (24 hours vs passing of one night), rather than erroneous recall. When the divergence between answers is one day or less, tests using calendar date and reported days since last menses are expected to produce very similar results, due to the similarity between estimated risk of conception on consecutive days (Pearson correlation between Wilcox (2001) estimate for current day and next day: *r*(38) = .96, *p* < .001). [↑](#footnote-ref-1)
